# The Impact of Physical Therapy Rehabilitation on Pain and Function in a Patient With Cauda Equina Syndrome

**DOI:** 10.7759/cureus.28131

**Published:** 2022-08-18

**Authors:** Aditi Joshi, Neha Chitale, Pratik Phansopkar

**Affiliations:** 1 Physiotherapy, Ravi Nair Physiotherapy College, Datta Meghe Institute of Medical Sciences, Wardha, IND; 2 Musculoskeletal Physiotherapy, Ravi Nair Physiotherapy College, Datta Meghe Institute of Medical Sciences, Wardha, IND

**Keywords:** physiotherapy rehabilitation, lumbar fractures, implant removal surgery, cauda equina syndrome, low back pain

## Abstract

The human spine is a complex and robust structure. Injury to the spine may contribute to limitations in activities of daily living. In the lumbar and sacral regions, the nerve roots continue as the cauda equina. These nerves communicate with the lower limbs and pelvic organs by sending and receiving messages. Cauda equina syndrome is a rare but potentially life-threatening illness caused by spinal canal compression of the cauda equina. Cauda equina syndrome occurs when there is dysfunction of multiple lumbar and sacral nerve roots of the cauda equina. Here is a case of a 25-year-old male, who visited the hospital with complaints of low back pain, weakness in the bilateral lower limb and urinary incontinence. He had a history of a fracture at lumbar vertebrae 10 years ago for which internal fixation was done. The patient was diagnosed with cauda equina syndrome post investigations and underwent implant removal surgery. He was further referred to the physiotherapy department for management of the same.

## Introduction

In the lumbar and sacral regions, nerve roots continue as cauda equina. These nerves communicate with the lower limbs and pelvic organs by transporting signals. Cauda equina syndrome is a rare but potentially life-threatening illness caused by compression of the spinal canal at the level of cauda equina [1]. This condition is rare but can have many causes. Most commonly it results from a massive herniated disk in the lumbar region. Herniation of the disk can be due to strain or any traumatic injury. Other causes can be named due to spinal tumor, fracture in the lumbar vertebrae, or any spinal surgery after which such complications may occur [2]. Patient experiences low back pain, weakness in lower limbs either unilaterally or bilaterally, pain in the gluteal region, and urinary incontinence. All of these leads to restriction in the activities of daily living of the patient. Nearly half of all patients complain of low back pain, which makes it difficult for them to be in any position and reduces their quality of life [3]. According to a study conducted in the United States of America, around 160,000 persons suffer from spinal column damage each year. Fractures are classified as mild or major based on the extent of the damage and the risk of instability. Compression fractures, burst fractures, seat belt fractures, and dislocation fractures are among the major fractures. Immobilization, medications, and surgery are given as options for treating spinal fractures [4]. These fractures are usually fixed with internal fixators. In this case, a young male was reported with severe low back pain and implant removal surgery from lumbar vertebrae which was fixed ten years prior and now causing compression at the level of cauda equina. The patient was planned for multidisciplinary management and rehabilitation to attain functional activities and enhance his quality of life.

## Case presentation

A 25-year-old male student, visited the multi-specialty hospital with complaints of low back pain and bilateral lower limbs weakness, after falling on farms. History revealed that the patient had fracture at lumbar vertebrae ten years ago after falling from approximately 7 feet of height. He was taken to the hospital near his residence where the vertebrae were fixed with implants. Now, after undergoing investigations, it was revealed that there was a compression at the cauda equina level. The patient, also complained on numbness in both feet and intermittent urinary incontinence. Post investigations, implant removal surgery was planned.

Clinical findings

**Figure 1:**
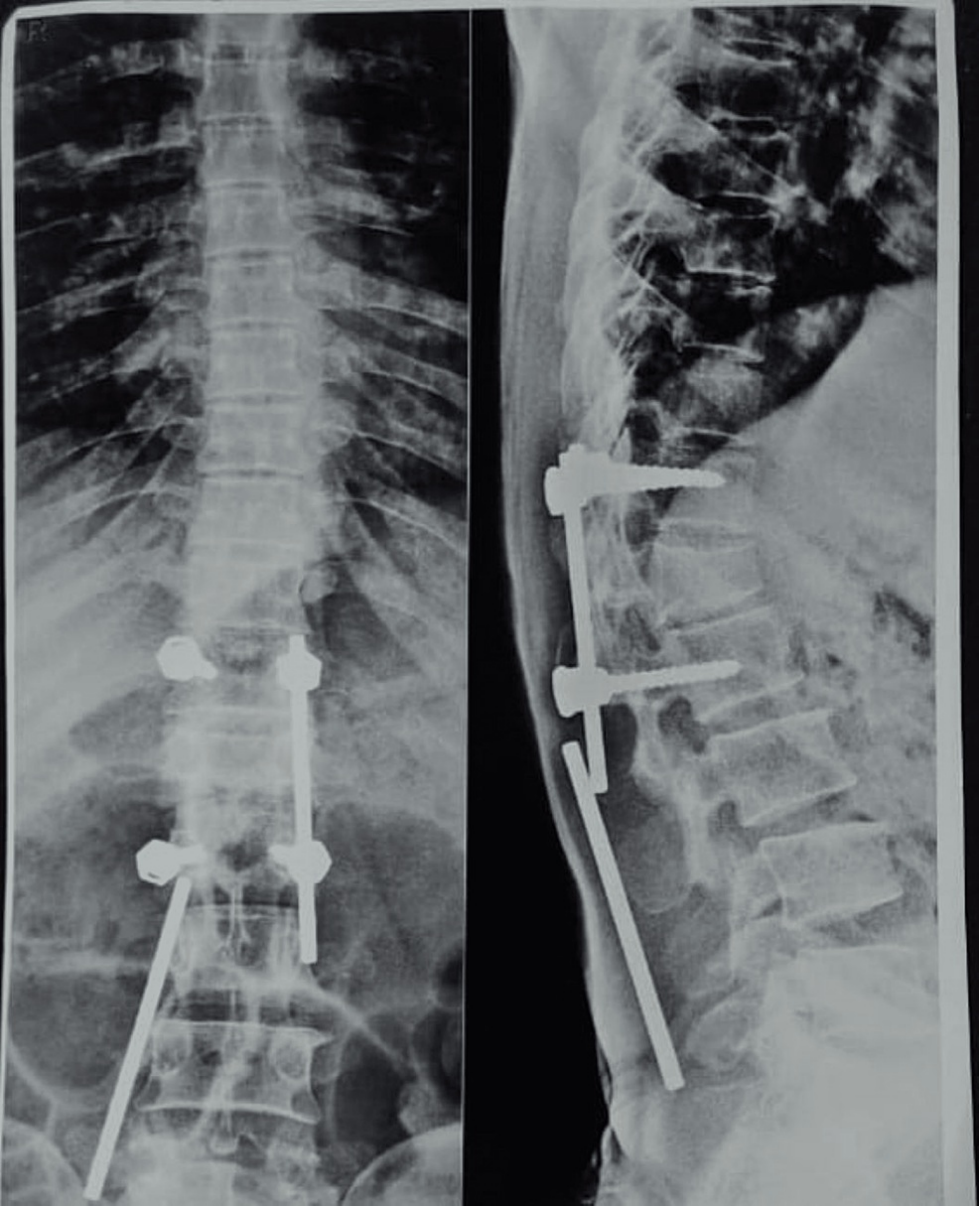
X-ray of vertebral column of the patient The X-ray image shows the fixation of implants in the vertebral column from posterior and lateral views.

Following patient’s informed consent, physical examination was conducted. On observation, the patient appeared to be awake, cooperative and well-oriented to time, place, and person. He was afebrile at time of examination. The pulse rate was 76 beats per minute and respiratory rate was 20 breaths per minute. Pain was prickling in nature and on numerical pain rating scale (NPRS) 7/10 during activity and 4/10 during rest. The radiological images showed the fixation of implants in the vertebral column, which can be seen in Figure 1. 

On examination, tenderness of grade II was present over the lumbar spine, the patient was found to have reduced range of motion of the left hip and knee joint due to weakness in the lower limbs (Table 1), and the strength in the lower limbs was reduced bilaterally. Manual muscle testing was performed on hip flexors, extensors, abductors, knee flexors, and extensors using the Medical Research Council scale. He was found to have normal lower limb reflexes and sensations to pinprick bilaterally in the L1-S1 dermatomes whereas rectal examination revealed a loss of sensation in the perianal area (S3, S4) with normal anal muscle tone. The McGill Pain Questionnaire, score was 58.

**Table 1:** Range of motion assessments of joints on the first day of rehabilitation

JOINT	MOVEMENTS	RANGE
Hip	Flexion	60
	Extension	25
	Abduction	30
Knee	Flexion	120

Therapeutic management 

The goal of physiotherapy and rehabilitation was to improve strength, fitness, joint mobility, proprioception, decreased pain, improve clinical symptoms to return to normal functioning, and improve the quality of life.

Phase 1 (week 1 to 2)

The patient was educated about his condition and the treatment protocol was explained to him. The initial goal was to relieve pain and promote relaxation. The pain was relieved with medications and proper postural guidance, in sitting and lying position, was given to the patient as it helped in relieving pain and providing relaxation. Active range of motion exercises for hip flexion, extension, abduction, and adduction; knee flexion and extension, and ankle dorsiflexion and plantar flexion exercises were taught. Active movements of both the upper extremities were taught to maintain the joint range. Isometric exercises to quadriceps and hamstring muscles were started by teaching static quadriceps and static hamstrings exercises in supine position. Along with these methods, core strengthening was initiated. In the beginning, only isometric exercises to abdominals and back muscles were taught. Active-assisted hip abduction was initiated, first, with five repetitions and in five sets with five seconds hold and gradually progressed to more number of repetitions for all exercises. It was increased to 10 repetitions in 10 sets with 10 seconds hold, and practicing twice a day. In the first phase of treatment, log rolling and bedside sitting were taught.

Phase 2 (week 2 - 4)

**Figure 2:**
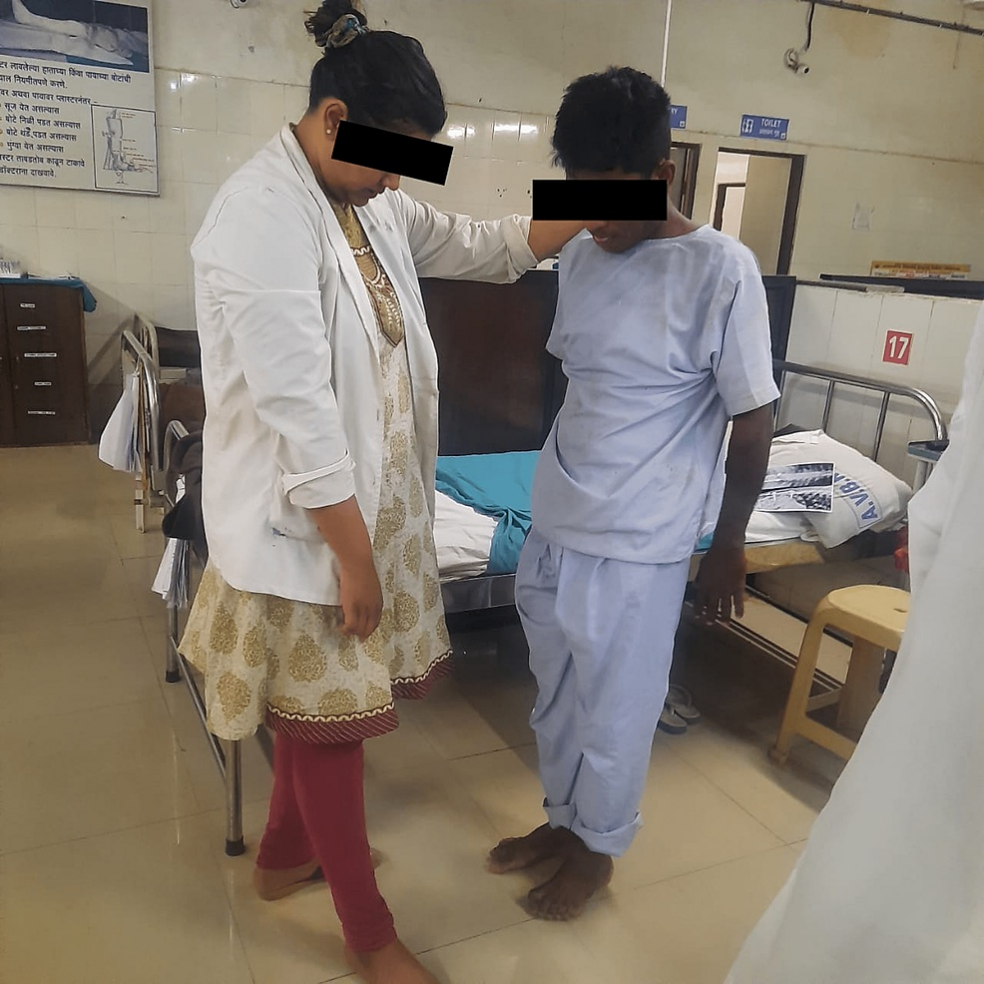
Balance training exercises Balance training exercises being taught to the patient in the standing position.

**Figure 3:**
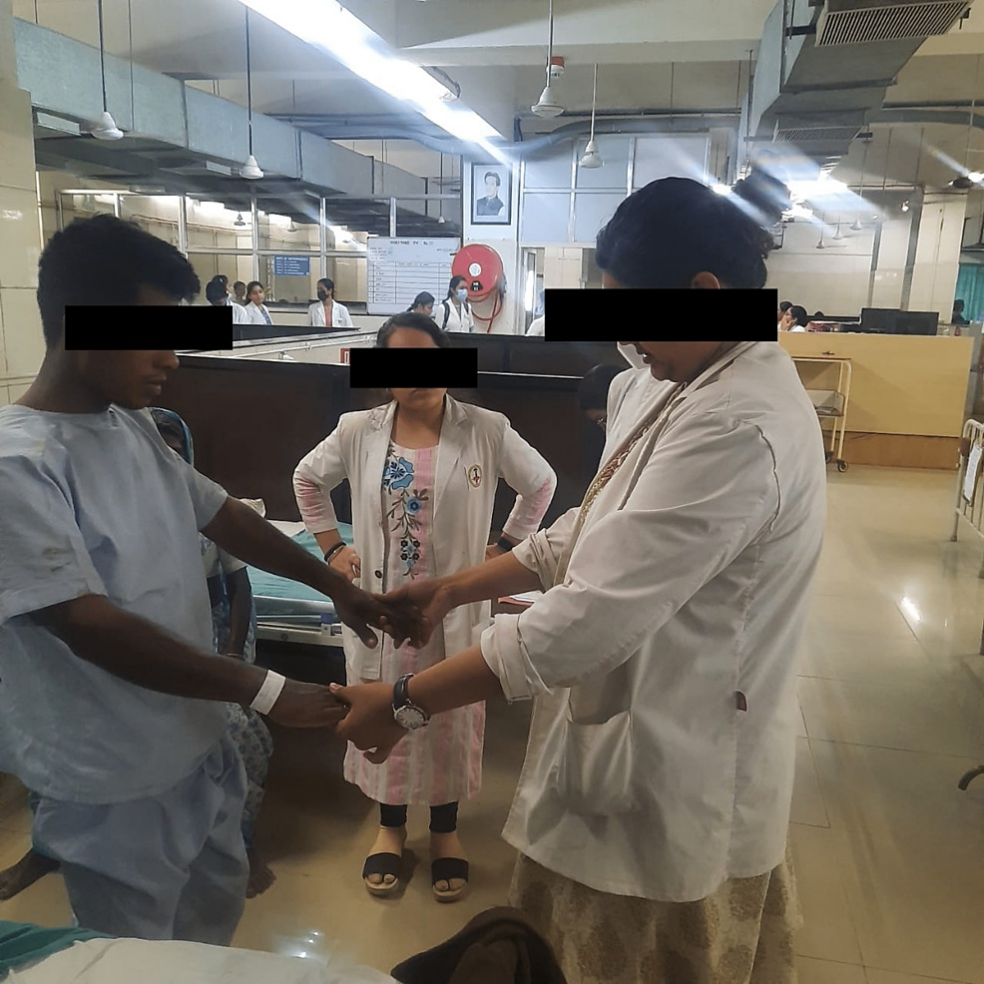
Gait training

This phase began with strength training to back, abdominals and resisted exercises to lower limbs and pelvic floor muscle exercises, which would help in treating urinary incontinence. Exercises in standing position were started along with balancing exercises as well as gait training to improve truncal control. This is seen in Figures 2-3, where the patient is being taught these activities. Ambulation with support was taught to the patient in this phase. The patient was instructed about basic ergonomics to be followed. A well-planned exercise program was planned for the patient, and he was instructed to continue the exercise program and follow ergonomic advice. 

Outcome measures

**Figure 4:**
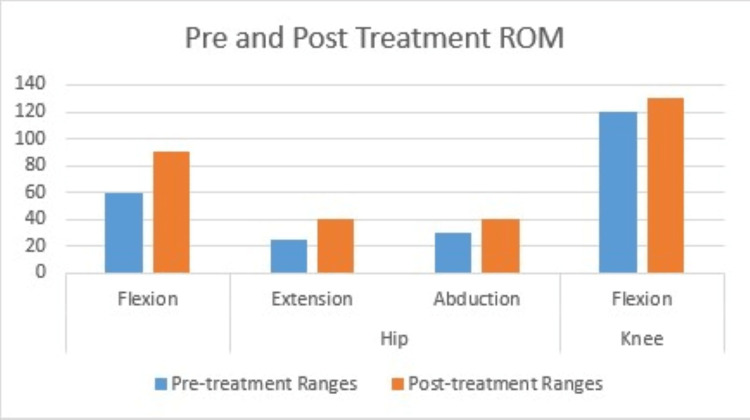
Comparison between pre- and post-treatment range of motion The x-axis represents the actions of muscles whereas the y-axis represents degrees of ROM. ROM: range of motion

The McGill Pain Questionnaire post-treatment score was 32. The comparison of pre- and post-treatment range of motion is shown in Figure 4.

## Discussion

Cauda equina syndrome is a mixture of neurologic symptoms and findings of different levels of severity, which include lower limb weakness, sensory loss, reduced reflexes, and, specifically, incontinence, with severe compression of the lumbar nerve roots [5]. Compression is mostly caused by degenerative stenosis or spondylolisthesis, however, fractures caused by posterior bone displacement or large extruded discs can also produce neurogenic claudication and eventually cauda equina syndrome after spine trauma [6]. The patient in this case complained of low back pain, radiating to the gluteal region on both sides, bilateral lower limbs weakness and urinary incontinence. After clinical examination, a conventional rehabilitation protocol was formed that aimed to reduce pain, increase strength in lower limbs, and eliminate the problem of urinary incontinence [7]. Strength training is essential for lower limbs as it reduces weakness and helps the patient to perform functional activities with ease. Core stabilization exercises are developed as exercise regimes for postural balance and play a key role in trunk control in static and dynamic postures. In this case report, this approach has been shown to improve muscle imbalance and decrease pain in the low back [8,9]. When inner and outer core muscles work properly, segmental spinal stability is maintained, the spine and pelvic area are protected, and stress and load on lumbar vertebrae and intervertebral discs are decreased. The outer core muscles compensate for any dysfunction, such as a weak inner core. Although the major role of outer core muscles is mobility rather than stability, they can help with stability when faced with unexpected overload. Splinting can result in neuromuscular problems like muscular spasms, neural compression, and discomfort [10]. Active range of motion exercises are performed by the patients on their own and are highly beneficial. They not only help in maintaining ranges, but also improve blood circulation to various parts of the limbs, and prevent complications like bed sores and stiff joints due to immobilization [11]. Ambulation and gait training help in improving balance and regaining the patient's confidence which also leads to psychological satisfaction for the patient. All these exercises can be beneficial in planning a rehabilitation protocol for patients with cauda equina syndrome.

## Conclusions

Cauda equina syndrome is one of the complications seen often after traumatic injuries. It is a medical emergency and needs to be managed by a multidisciplinary team. A proper rehabilitation results in making the patient functionally able in performing activities of daily living with ease. Following four weeks of rehabilitation, in this case, there was a significant improvement in range of motion, muscle strength, pain reduction, and functional ability that helped in preventing complications. This case report establishes a properly structured and comprehensive rehabilitation protocol for dealing with cauda equina syndrome with low back pain.
